# An online operational rainfall-monitoring resource for epidemic malaria early warning systems in Africa

**DOI:** 10.1186/1475-2875-4-6

**Published:** 2005-01-21

**Authors:** Emily Grover-Kopec, Mika Kawano, Robert W Klaver, Benno Blumenthal, Pietro Ceccato, Stephen J Connor

**Affiliations:** 1 International Research Institute for Climate Prediction (IRI), The Earth Institute at Columbia University, Monell, Lamont Campus, 61 Route 9W, Palisades, New York 10964-8000, USA; 2 Public Health Mapping Group and Geographic Information Systems, Communicable Diseases (CDS), World Health Organization, 20, Avenue Appia, 1211 Geneva 27, Switzerland; 3 United States Geological Survey, EROS Data Center, Sioux Falls, South Dakota 57198, USA

## Abstract

Periodic epidemics of malaria are a major public health problem for many sub-Saharan African countries. Populations in epidemic prone areas have a poorly developed immunity to malaria and the disease remains life threatening to all age groups. The impact of epidemics could be minimized by prediction and improved prevention through timely vector control and deployment of appropriate drugs. Malaria Early Warning Systems are advocated as a means of improving the opportunity for preparedness and timely response.

Rainfall is one of the major factors triggering epidemics in warm semi-arid and desert-fringe areas. Explosive epidemics often occur in these regions after excessive rains and, where these follow periods of drought and poor food security, can be especially severe. Consequently, rainfall monitoring forms one of the essential elements for the development of integrated Malaria Early Warning Systems for sub-Saharan Africa, as outlined by the World Health Organization.

The Roll Back Malaria Technical Resource Network on Prevention and Control of Epidemics recommended that a simple indicator of changes in epidemic risk in regions of marginal transmission, consisting primarily of rainfall anomaly maps, could provide immediate benefit to early warning efforts. In response to these recommendations, the Famine Early Warning Systems Network produced maps that combine information about dekadal rainfall anomalies, and epidemic malaria risk, available via their Africa Data Dissemination Service. These maps were later made available in a format that is directly compatible with HealthMapper, the mapping and surveillance software developed by the WHO's Communicable Disease Surveillance and Response Department. A new monitoring interface has recently been developed at the International Research Institute for Climate Prediction (IRI) that enables the user to gain a more contextual perspective of the current rainfall estimates by comparing them to previous seasons and climatological averages. These resources are available at no cost to the user and are updated on a routine basis.

## Introduction

It is estimated that more than 110 million Africans live in areas prone to epidemics of malaria. Populations in these areas are infrequently challenged by malaria and, therefore, do not fully develop acquired immunity. As a result, the disease remains life threatening to all age groups. The impact of malaria epidemics could be greatly reduced by timely detection or, ideally, by prediction and prevention through vector control and deployment of appropriate drugs [1].

Rainfall is recognized as one of the major factors influencing variability in malaria transmission in warm semi-arid and desert-fringe areas of the Sahel, the Greater Horn and Southern Africa. Explosive epidemics may occur in these regions after excessive rains, usually with a lag-time of several weeks during which time mosquito vector populations and malaria infections increase rapidly. Epidemics can be especially severe among communities stressed by recent periods of drought and poor food security. Recent research has found that rainfall estimates would be able to provide useful epidemic early warning information, even in highland-fringe settings, such as those in Kenya and Ethiopia, where temperature is also an important limiting factor for the development of the malaria parasite [2-4]. Consequently, rainfall monitoring forms one of the essential elements for the development of integrated Malaria Early Warning Systems for sub-Saharan Africa, as outlined by the World Health Organization [5,6].

During a meeting of the Roll Back Malaria Technical Resource Network on Prevention and Control of Epidemics (RBM-TSN) it was decided that immediate benefit could be realized from the routine availability of a simple indicator of changes in epidemic risk in these regions of marginal transmission [7]. The indicator was to be based on the difference between current rainfall (derived from meteorological satellite estimates) and the expected (average) for the particular time of the year; results were to be made available on the internet in a frequently updated format. The purpose was to provide timely alerts to malaria control programme staff and RBM partners working in areas of increased epidemic risk [8].

The following article provides an update on existing online rainfall monitoring resources and a description of a new tool that has been developed to address remaining needs of the malaria control community

## Discussion

**Figure 1:**
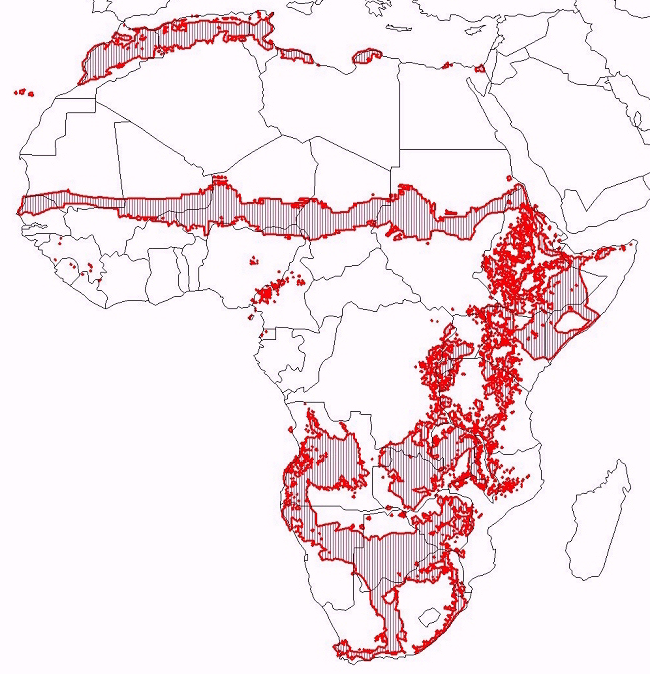
Epidemic risk zones in Africa (adapted from [1]).

Following discussion among members of the RBM-TSN a consensus map of epidemic risk zones was produced [7]. The map, shown in Figure 1, was used as a mask to exclude areas where malaria transmission is considered absent or endemic, as opposed to epidemic. This mask is based purely on climatic constraints to malaria transmission, and does not yet account for areas in the northern and southern margins of the continent where control has eliminated malaria risk.

**Figure 2:**
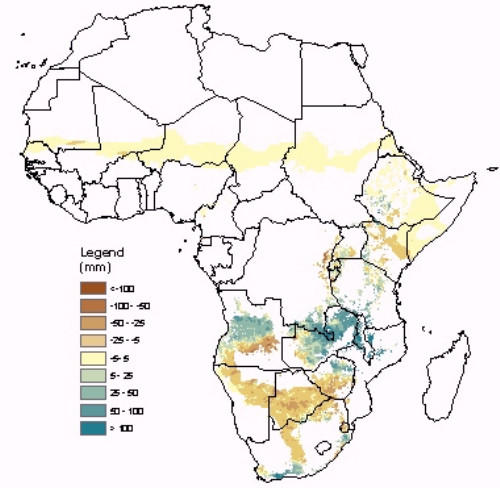
Rainfall Anomalies in Zones with Malaria Epidemic Potential: 21–31 December 2004

The epidemic risk map was then combined with rainfall anomaly data (i.e., the difference between observed rainfall and the expected, i.e. average, rainfall for a particular time of the year) to provide a simple indicator of changes in risk in epidemic prone areas. Figure 2 illustrates the resulting dekadal (i.e., ~10-daily) rainfall anomaly maps, which are updated approximately every 10 days, and have been available in experimental form through the Africa Data Dissemination Service (ADDS) since June 2002. The ADDS is an operational part of the Famine Early Warning Systems Network (FEWS NET) which is maintained by the United States Geological Survey (USGS) and supported by the U. S. Agency for International Development (USAID). The maps can be accessed at:  under "RFE Anomaly – Malaria", where the anomaly map for the most recent dekad is displayed along with documentation on how the map is produced. The existence of this online monitoring resource was publicized and their use and validation by control services and researchers was encouraged [8]. The rainfall estimate data underlying these maps was tested against laboratory-confirmed malaria incidence figures for selected districts in Southern Africa, where they showed a good association [9].

In the year following the launch of the ADDS dekadal rainfall anomaly maps, WHO commissioned field visits to a number of epidemic prone countries to evaluate whether the National Malaria Control Programmes were aware of this resource and how useful they considered it may be for their efforts. Sudan, Uganda, Niger, Mali and Burkina Faso received field visits. In general, all of the control programmes had been aware of the rainfall anomaly maps, but only those in Uganda and Sudan had monitored them regularly during the previous year. The control programmes in the Sahelian countries did not agree with the epidemic risk zone used in the mask because their recent experience was that epidemic outbreaks had occurred beyond the northern boundary of the epidemic risk zone. This was also partly true in Sudan where epidemic outbreaks have been known to occur along the Nile River margins in the northern half of the country. Uganda's malaria control programme, however, had found the maps to be reasonably accurate and a useful monitoring resource. Further dialogue with malaria control programmes in West Africa and Southern Africa also raised the point that a single dekadal rainfall anomaly map could raise an alert; when in fact the rainfall levels were not abnormally high – but just 10 days earlier than 'normal'. This suggested that additional information about the temporal distribution of rainfall was necessary.

**Figure 3:**
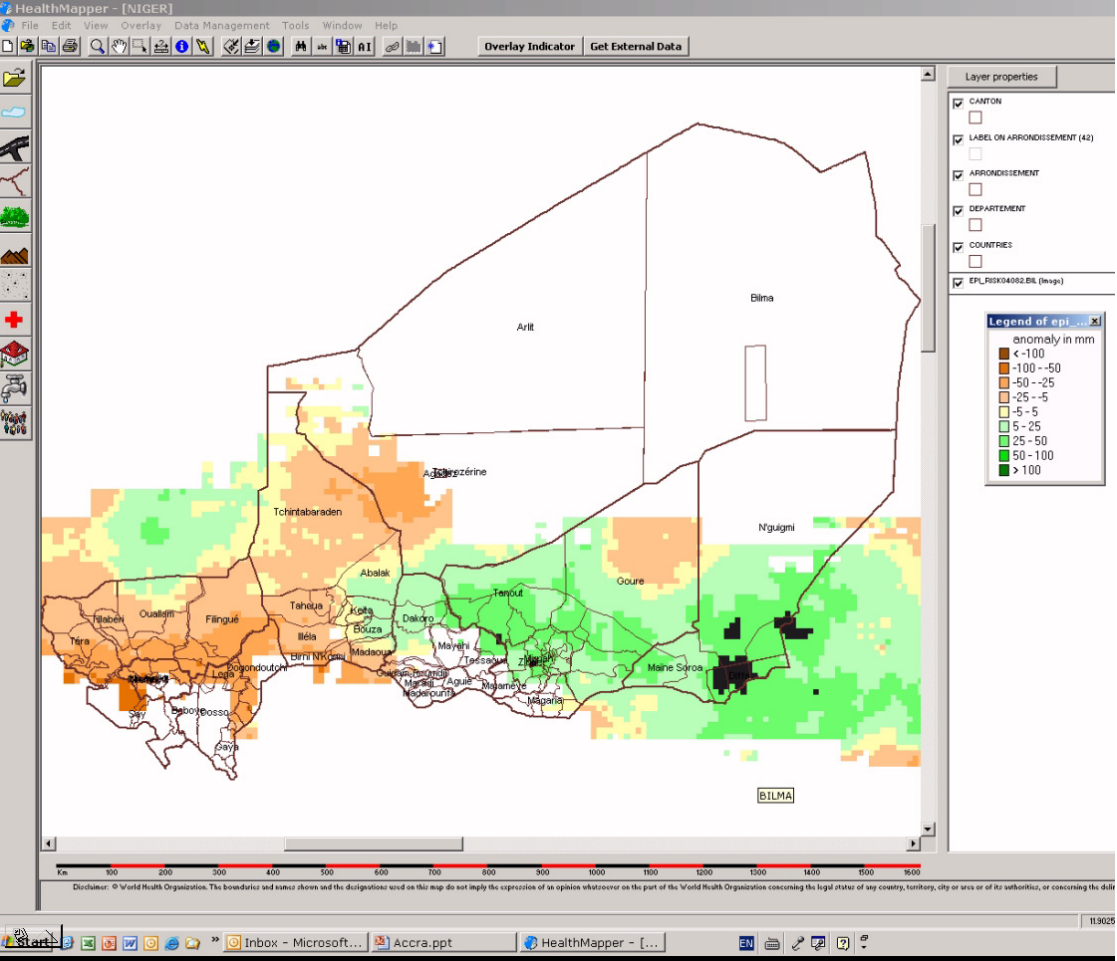
10 daily rainfall anomaly map for Niger viewed in the HealthMapper

In order to respond to these issues, USGS and WHO-HealthMapper agreed to collaborate on the development of the dekadal anomaly maps in a format which could be downloaded, viewed and archived by surveillance staff directly in HealthMapper, a basic mapping and surveillance software developed by WHO's Communicable Disease Surveillance and Response Department . In addition to the most recent map, it is possible to download the dekadal maps for the previous six months and begin to construct a seasonal time series. The integration of the rainfall anomalies maps within HealthMapper also allowed the users to improve their analyses by combining ancillary data related to malaria directly on top of the rainfall anomalies maps for their country. Figure 3 provides an example for Niger.

**Figure 4:**
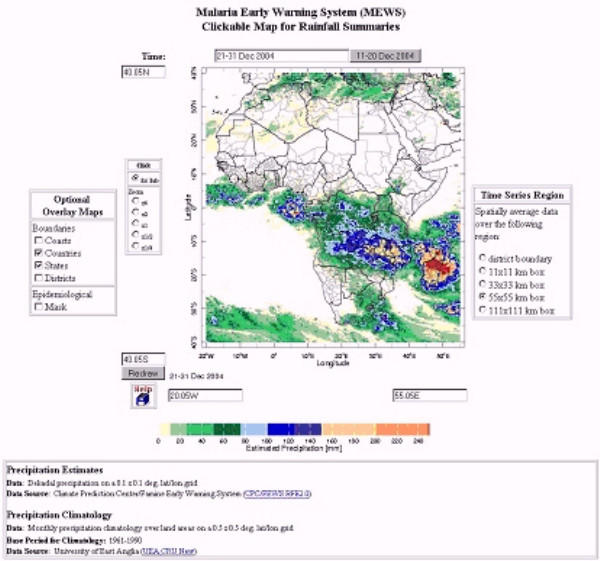
MEWS 'Clickable Map' for Rainfall Monitoring: 21–31 December 2004

**Figure 5:**
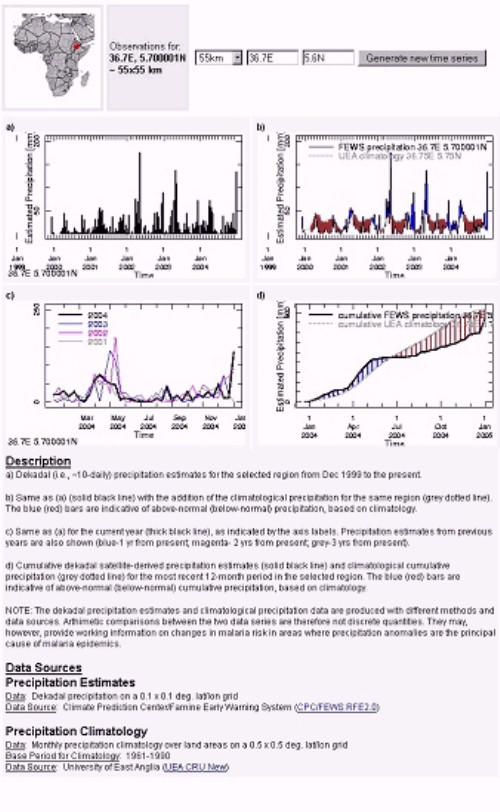
Summary information on current rainfall/seasonal development and expected rainfall for location of interest.

Staff working at the International Research Institute for Climate Prediction (IRI) have since developed a web-based Malaria Early Warning System (MEWS) interface that enables the user to gain a broader contextual perspective of the current rainfall season by comparing it to previous seasons and climatological averages. The interface is in the IRI Data Library and takes the form of an online 'clickable map' of Africa: . It displays the most recent dekadal rainfall map (Figure 4) over which national and district administrative boundaries and the epidemic risk zone can be overlaid (in this case as a guide rather than an absolute mask which may have excluded districts of local interest). These visual features can be toggled on or off and the user can zoom in to any region for more clarity. The user can 'zoom' into a more localized region of interest. Dekadal rainfall can be spatially-averaged over a variety of user-selected areas, including administrative districts and 11 × 11 km, 33 × 33 km, 55 × 55 km and 111 × 111 km boxes. Upon the selection of this sampling area and a specific location of interest (by a click on map at the location of interest), four time-series graphs are generated (Figure 5). These time-series provide an analysis of recent rainfall with respect to that of recent seasons and the overall climatology. A description of the time-series figures, the data used and its source is also provided.

## Conclusions

Access to frequently-updated rainfall information is an important requirement for the development of integrated early warning systems for malaria and other climate sensitive diseases [6,10]. These operational rainfall monitoring tools have been developed primarily for application in warm semi-arid regions where rainfall anomalies are the main determinant of epidemic outbreaks, however they may also be an important information source for highland-fringe epidemic settings. They are offered as an experimental resource for testing within MEWS applications in Africa – and may be modified in future in response to user feedback and further evaluation.

While it is recognized that much of Africa does not (yet) have easy access to the internet, email is becoming more prevalent and it is now relatively easy for regional support centres, such as the WHO-Intercountry Programme for Malaria Control in Southern Africa, to prepare bulletins with malaria relevant climate data for distribution by email and courier to district health teams in epidemic prone areas as part of an overall MEWS process [11].
